# Clinical Survey of Dengue Virus Circulation in the Republic of Djibouti between 2011 and 2014 Identifies Serotype 3 Epidemic and Recommends Clinical Diagnosis Guidelines for Resource Limited Settings

**DOI:** 10.1371/journal.pntd.0004755

**Published:** 2016-06-20

**Authors:** Erwan Le Gonidec, Marianne Maquart, Sandrine Duron, Hélène Savini, Geraldine Cazajous, Pierre-Olivier Vidal, Marie-Caroline Chenilleau, Jean-Baptiste Roseau, Alain Benois, Céline Dehan, Jeffrey Kugelman, Isabelle Leparc-Goffart, Serge Védy

**Affiliations:** 1 Emergency Department, Bouffard Military Hospital, Djibouti, Republic of Djibouti; 2 French National Reference Centre for Arbovirus—Armed Forces Biomedical Research Institute, Marseille, France; 3 French Armed forces Centre for Epidemiology and Public Health (CESPA), GSBdD Marseille Aubagne, Marseille, France; 4 Department of Infectious and Tropical Diseases, Laveran Military Teaching Hospital, Marseille, France; 5 Department of Medicine, Bouffard Military Hospital, Djibouti, Republic of Djibouti; 6 Department of Anesthesiology and Intensive Care, Bouffard Military Hospital, Djibouti, Republic of Djibouti; 7 Department of Biology, Bouffard Military Hospital, Djibouti, Republic of Djibouti; 8 U.S. Army Medical Research and Materiel Command, Fort Detrick, Maryland, United States of America; Florida Department of Health, UNITED STATES

## Abstract

Dengue virus is endemic globally, throughout tropical and sub-tropical regions. While the number of epidemics due to the four DENV serotypes is pronounced in East Africa, the total number of cases reported in Africa (16 million infections) remained at low levels compared to Asia (70 million infections). The French Armed forces Health Service provides epidemiological surveillance support in the Republic of Djibouti through the Bouffard Military hospital. Between 2011 and 2014, clinical and biological data of suspected dengue syndromes were collected at the Bouffard Military hospital and analyzed to improve Dengue clinical diagnosis and evaluate its circulation in East Africa. Examining samples from patients that presented one or more Dengue-like symptoms the study evidenced 128 Dengue cases among 354 suspected cases (36.2% of the non-malarial Dengue-like syndromes). It also demonstrated the circulation of serotypes 1 and 2 and reports the first epidemic of serotype 3 infections in Djibouti which was found in all of the hospitalized patients in this study. Based on these results we have determined that screening for Malaria and the presence of the arthralgia, gastro-intestinal symptoms and lymphopenia < 1,000cell/ mm3 allows for negative predictive value and specificity of diagnosis in isolated areas superior to 80% up to day 6. This study also provides evidence for an epidemic of Dengue virus serotype 3 previously not detected in Djibouti.

## Author Summary

Dengue virus is emerging worldwide, however, little is known about the burden of Dengue in Africa. Effectively, only sporadic cases and few epidemics have been reported in the last 30 years. This descriptive study reports clinical and biological data of Dengue-suspected cases analyzed in the Bouffard military hospital of Djibouti from 2011–2014. It also confirms the circulation of Dengue virus serotypes 1 and 2 and reports the first epidemic of Dengue virus serotype 3 infections in Djibouti. Directions for diagnosis are offered to practitioners working in resource limited settings and dealing with a Dengue-like syndrome of less than seven days and negative for malaria.

## Introduction

With 3,900 million people exposed in 128 countries, the Dengue virus (DENV) is the most common arboviral disease in the world. Every year, 50 to 100 million people are infected and at least 30,000 people die [1–3]. Four antigenically distinct serotypes (DENV1-4) circulate simultaneously in endemic countries [4]. The virus is endemic globally in tropical and sub-tropical regions. Primarily in Asia, South and Central America [5], however, reports on Dengue circulation are increasing in Africa [6, 7]. Although the number of epidemics due to the four serotypes is markedly increased in East Africa [8] and to a lesser extent in West Africa [9], the number of cases reported in Africa (16 million infections) remain at very low level compared to Asia (67 million infections) and nearly equivalent to that of the Americas (13 million infections) [10]. The first epidemic of DENV in Djibouti was reported in 1991–1992 with 12,000 cases of serotype 2 [11]. Introduction of DENV1 in Djibouti was detected in 1998 with sporadic cases until 2000 [7]. Between 2000 and 2002, 185 cases of Dengue virus were detected by serology with only 6 identified as serotype 1 by virus isolation [12].

Due to the continuous geographic expansion of Dengue disease, the World Health Organization implemented a surveillance program [3]. The French Armed Forces Health Service (FAFHS) participates in this program through its overseas sentinel network [13]. In the Republic of Djibouti, the public health system relies on the activity of the medical and surgical hospital Bouffard (HMC Bouffard). Due to its diversified technical equipment, HMC Bouffard cares for the overseas community and the Djiboutian people

This study provides an analysis of epidemiological, clinical and biological data related to suspected and confirmed cases of Dengue managed at HMC Bouffard between 2011 and 2014. Based on the observed symptoms, the complete blood count data, and the biological diagnosis, this study aims to help practitioners working in isolated areas to establish Dengue etiology in resource limited settings.

## Materials and Methods

### Study presentation

This retrospective study presents the clinical and biological analysis results of Dengue-suspected cases managed at HMC Bouffard between January 2011 and May 2014. Dengue-like cases were defined when patients showed one or more of the following signs: fever, arthralgia, headache, myalgia, gastro-intestinal symptoms, hemorrhagic syndrome and visceral organ failure. Every patient was tested for Malaria using the HRP-2 test (Core Malaria, Core Diagnostics, Birmingham, UK) and the QBC method. In this study, we considered as Dengue-suspected cases patients presenting a Dengue-like syndrome and testing negative for malaria. These patients were recruited through the emergency department of the hospital (admissions to the hospital for medical checkups due to fever represent 6% of the patients) or directly at the medical laboratory when they came with a medical prescription issued by civilian or military physicians practicing in Djibouti.

A case of dengue was confirmed (DENV-POS) when a suspected patient test produced at least one positive result for one of the following diagnostic tests: NS1 Antigen (NS1 Ag), anti-Dengue Immunoglobulin M (IgM) or real-time polymerase chain reaction (RT-PCR). Isolation of Flavivirus specific IgG alone is insufficient for a diagnosis of recent DENV infection.

Suspected patients who had negative results for the three DENV diagnostics were considered as negatives (DENV-NEG).

For each suspected case, an epidemiological form (C11 syndrome « dengue-like ») provided by the FAFHS was filled in to gather demographic, clinical and epidemiological data. Biological sampling was done in order to achieve a complete blood count and the confirmatory diagnosis of Dengue cases.

The following data come from the C11 forms: number of Dengue cases among the suspected cases, periodicity, serotype, clinical signs, platelet and lymphocyte counts at the physician visit.

### Ethics statement

Diagnosis for arboviruses for all the French military hospitals, and for the French soldiers in Africa is assessed by the French National Reference Center (NRC) for Arboviruses belonging to the French Armed Forces Biomedical Research Institute. As the Reference Center, arbovirus diagnosis was realized only when all the required information was provided: clinical and biological data, travel history and date of onset of symptoms. All the data presented in this paper were used for the routine diagnosis.

### Complete blood count

For each sample, a complete blood count was automatically performed (Pentra 120, ABX company) at the Bouffard hospital laboratory. No follow-up of the platelet and lymphocyte counts were performed.

### Detection of dengue virus and typing

All the samples collected in the range of 0 to 7 days after onset of symptoms were tested in order to detect DENV viral RNA and viral NS1 Ag.

The NS1 Ag was tested with the SD BIOLINE Dengue Duo rapid test (NS1 Ag + IgG/IgM) (Standard Diagnostics, Inc, Republic Of Korea) according to the manufacturer's instructions. It is a rapid, in vitro immunochromatographic, one-step assay designed to detect both DENV NS1 antigen and IgG/IgM antibodies to DENV in human serum, plasma or whole blood.

Viral RNA was extracted from 140μl of serum using QIAamp Viral RNA Mini Kits (Qiagen, Germany) by the French NRC for arboviruses. Five systems of real-time RT-PCR assays were used to detect all viral strains and identify the 4 serotypes, as previously described by Leparc-Goffart et al. [14].

### Serological analysis

Each sample collected at the HMC Bouffard was analyzed with the rapid test SD BIOLINE Dengue Duo (NS1 Ag + IgG/IgM).

Concurrently, each sample collected at least 5 days after the onset of symptoms was serologically analyzed (IgM and IgG) to detect DENV, Chikungunya, West Nile and Rift Valley Fever infections. In-house IgM antibody capture enzyme-linked immunosorbent assay (MAC-ELISA) and IgG indirect ELISA were used to detect IgM and IgG antibodies to DENV [15].

### Statistical analyses

After a first step focused on the descriptive analysis of the study population, platelet and lymphocyte counts were compared as a function of Dengue status (DENV-POS versus DENV-NEG) and then among DENV serotypes in DENV-POS patients. The statistical tests used were non-parametric tests, as conditions for application of parametric tests were not fulfilled. A Mann-Whitney test was used to compare the median counts as a function of the dengue status and a Kruskal-Wallis test was used to compare the median counts as a function of the dengue serotype.

Then, a univariate analysis was performed to determine the relationship between the different symptoms or clinical signs and the Dengue diagnosis. The statistical tests used were the Chi-squared test, or the Fisher’s exact test when the conditions for application of the Chi-squared test were not fulfilled.

A multivariate analysis was finally performed to determine the main independent symptoms or clinical signs associated with Dengue diagnosis. All the independent variables achieving a p-value ≤ 0.20 at the univariate stage were introduced in the multivariate analysis model. Odds ratios were obtained using logistic regression models. Finally, the same analysis (univariate analysis followed by a multivariate analysis) was performed for the DENV serotype among the DENV-POS patients, using multinomial regression models. The statistical significance was defined as a p-value <0.05. All data were analyzed using the Stata Statistical Software v12 (Statacorp, USA).

## Results

### Progression of the dengue cases in Djibouti between 2011 and 2014

**Fig 1:**
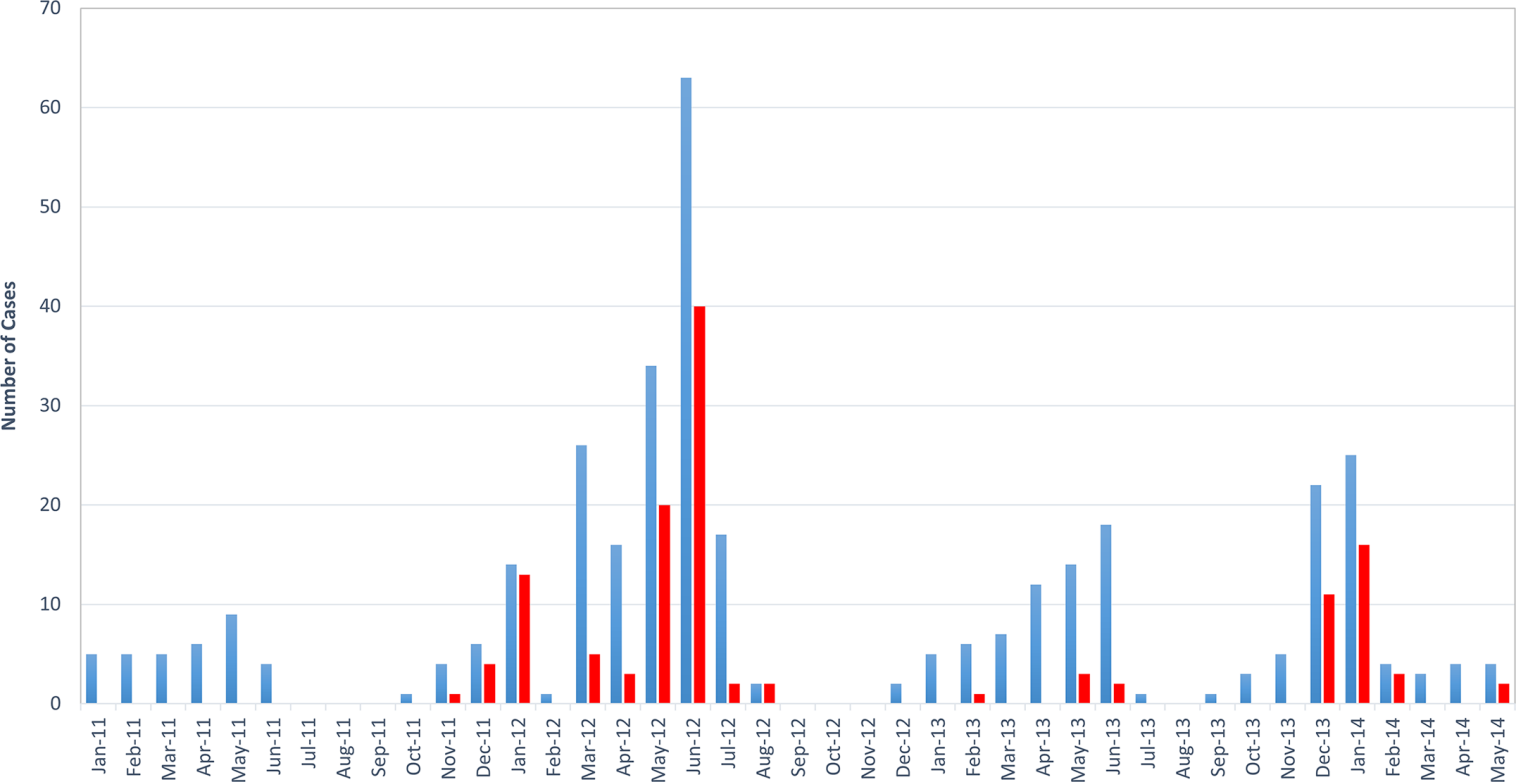
Suspected and confirmed cases of Dengue from January 2011 to May 2014 by month. Suspected cases are represented in blue and confirmed cases in red.

Between 2011 and 2014, 354 Dengue-suspected cases, presenting with one or more Dengue-like symptoms, were cared for in HMC Bouffard (Fig 1). A total of 128 dengue cases, as well as one West Nile virus case, one Rift Valley fever case and one imported Zika virus case from French Polynesia were diagnosed and confirmed by the Arboviruses NRC. Most of the DENV-POS patients were male (66%). Military personnel were highly represented, comprising more than 30% of the dengue positive cases. The remaining 70% corresponded to the military personnel’s family, the expatriate population and some Djiboutian civilians.

Twelve cases presented only a positive serology (presence of IgM and anti-flavivirus IgG antibodies), 38 cases were only NS1-positive. A total of 78 dengue cases were diagnosed by PCR.

**Fig 2:**
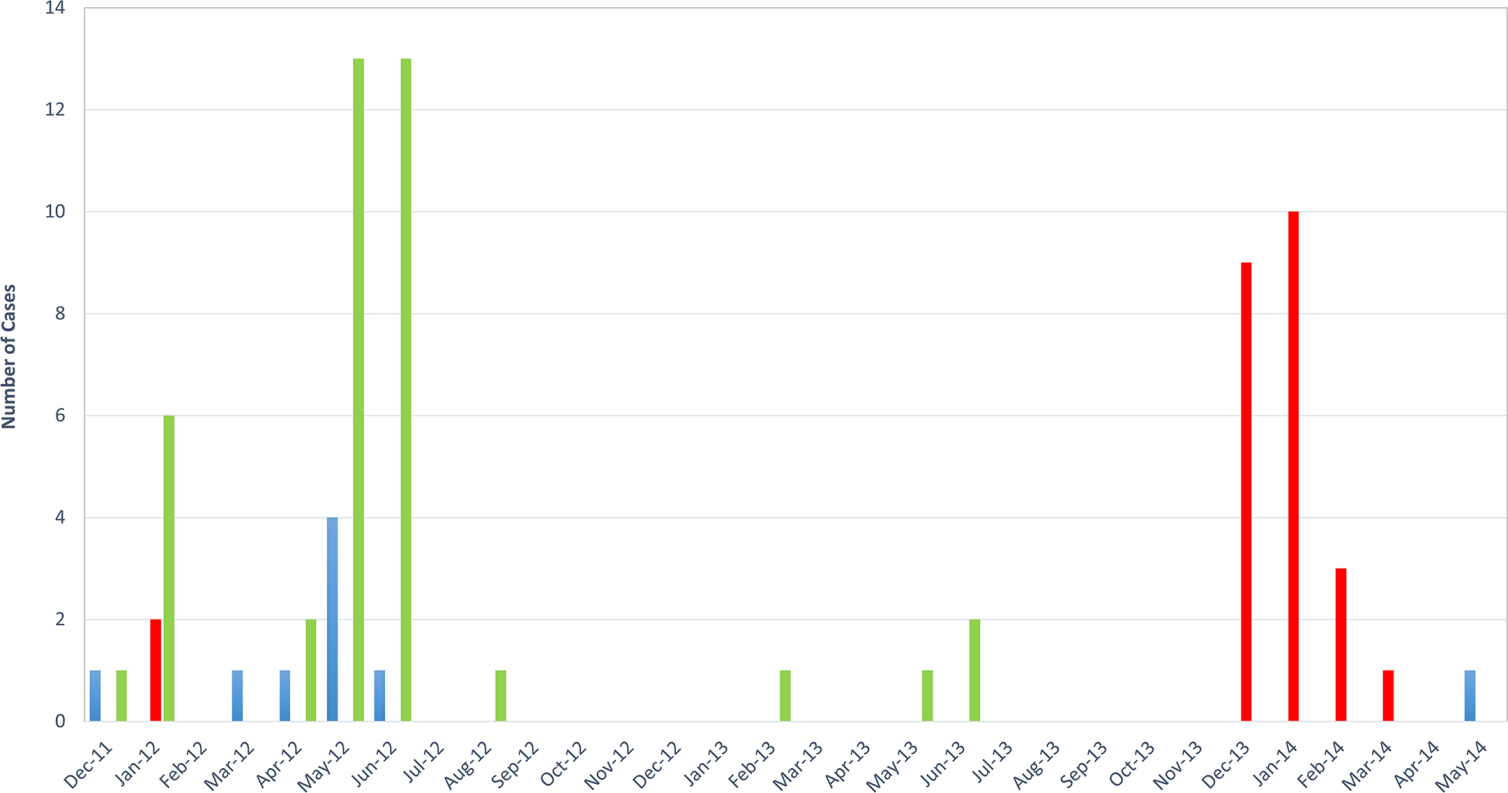
Number of cases of Dengue virus serotypes from December 2011 to May 2014 by month. Blue bars represented serotype 1, red bars the serotype 2 and green bars serotype 3.

Most of the cases detected by PCR (74/78) could be serotyped: there were 9 cases of DENV1 (12%), 25 cases of DENV2 (34%) and 40 cases of DENV3 (54%) (Fig 2).

Cases of DENV1 occurred between November 2011 and June 2012, with a recurrence in June 2014. The epidemic of DENV3 began in December 2011, reached an epidemic peak during the summer of 2012 and declined the winter of 2012–2013. A few cases of DENV3 were reported during the spring of 2013. Cases of DENV2 occurred during a short period, in epidemic form, from December 2013 to March 2014, with 23 cases reported in 4 months.

### Analysis of symptoms in DENV-POS and DENV-NEG patients, depending on serotypes

Clinical signs were studied from 98 DEN-POS files (i.e. 77% of the positive cases). The other files were not taken into account as patients were addressed without clinical data to the hospital laboratory by a physician from the inner-city. Among the analyzed cases, 98% suffered from fever, 89% from arthralgia, 75% from headache, 39% from gastrointestinal symptoms such as nausea and vomiting, 35% from retro-orbital pain and 22% from a cutaneous rash. No atypia was identified.

Ten patients were admitted to the hospital, i.e. 7.8% of the DENV-POS patients. Among them, 6 could be serotyped and were infected with the DENV-3 virus. In 6 cases, the criteria for hospitalization were a dehydration caused by digestive disorders and hyperthermia. Two female patients were admitted in the department of intensive care with severe Dengue: an 8-year old girl for a Dengue Hemorrhagic Fever, in a state of shock and with multiple organ failure and a 50-year old woman, without any comorbidity, for an isolated acute pulmonary edema, without any hemorrhagic sign. No deaths were reported.

**Fig 3:**
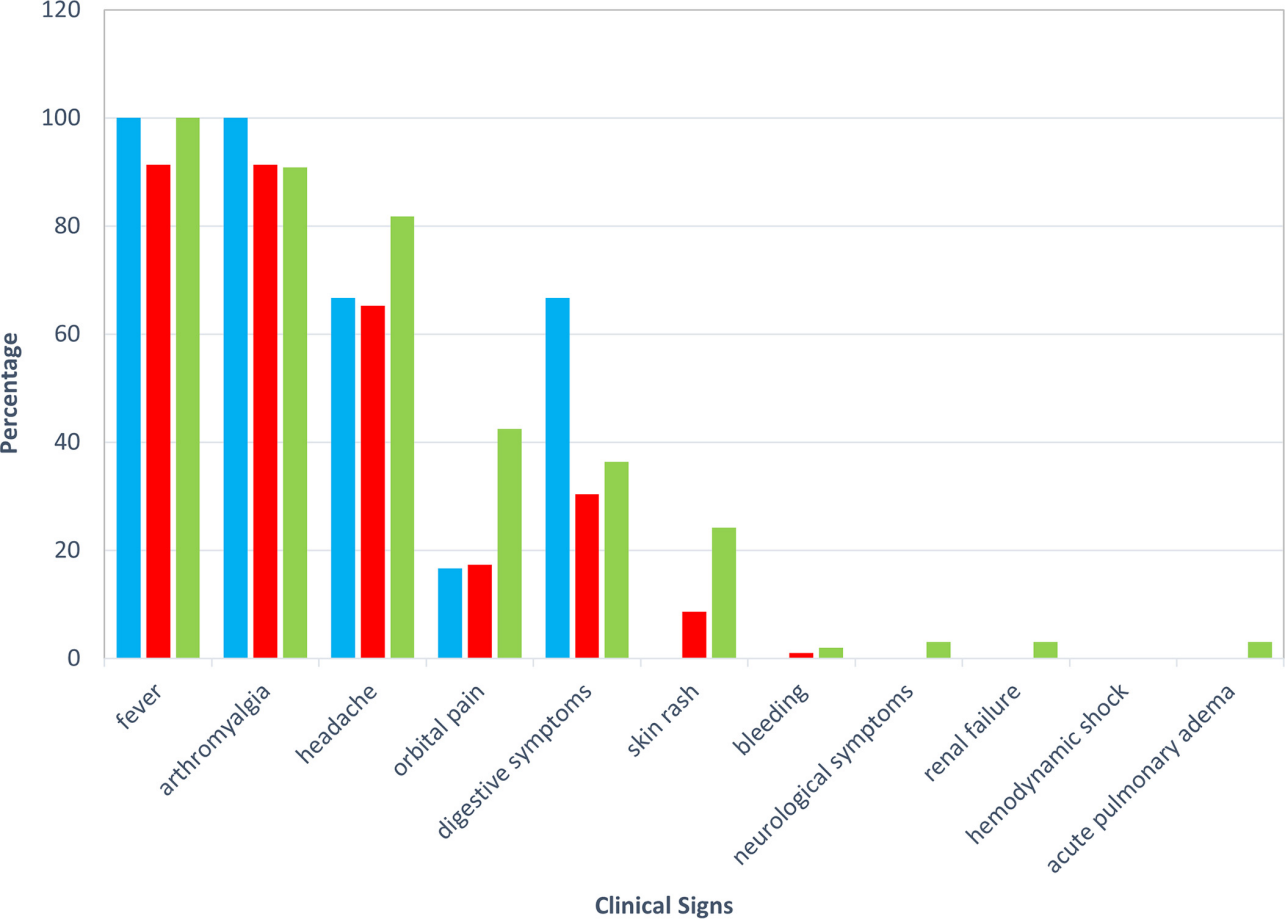
Clinical signs observed for each serotype of Dengue virus. Blue bars represented serotype 1, red bars serotype 2 and green bars serotype 3.

After univariate analysis according to Dengue status (comparison of DENV-POS and DENV-NEG patients' clinical signs), the presence of arthralgia was the only factor significantly associated to Dengue disease (p = 0.007). This association persisted after multivariate analysis, as patients presenting arthralgia were 2.5 times more likely to be DENV-POS (95% confidence interval of adjusted OR: 1.2–5.2), adjusted for the presence of gastrointestinal symptoms (adjusted OR: 1.6; 95% CI: 0.9–2.7) (Table 1). Presence of retro-orbital pain and skin rash seemed to be more frequent for DENV3 cases (Fig 3). However, no statistically significant association between these symptoms and serotypes was found (respectively p = 0.13 and p = 0.39).

**Table 1:** Clinical signs and symptoms according to Dengue diagnosis (Dengue positive vs Dengue negative patients)—Results of univariate and multivariate analyses. p-values were obtained using likelihood ratio tests.

	Univariate analysis			Multivariate analysis		
	ORc	95% CI	p-value	ORa	95% CI	p-value
Fever	2.4	0.5; 11.7	0.26			
Arthralgia	2.6	1.3; 5.4	0.009	2.5	1.2; 5.2	0.01
Headache	1.5	0.9; 2.7	0.14			
Retro orbital pain	1.4	0.8; 2.4	0.22			
Gastro intestinal symptoms	1.6	1.0; 2.8	0.07	1.6	0.9; 2.7	0.10
Skin rash	1.2	0.7; 2.3	0.47			

ORc: crude odds ratio; ORa: adjusted odds ratio; 95% CI: 95% confidence interval

### Lymphocyte and platelet counts in DENV-POS and DENV-NEG patients, depending on serotypes

The lymphocyte count at admission was significantly lower in DENV-POS patients than in DENV-NEG patients (respectively 700 cells/mm3 vs 1,330 cells/mm3) (p<0.001) (Table 2). The lowest lymphocyte count was measured in a DENV-POS patient being tested of two days after the onset of the symptoms at 200 cells/mm3. In the patients being tested after the 5th day of clinical evolution, the lymphocyte count returned to basal levels. Finally, the median lymphocyte count at admission was significantly lower in serotype-3 than in serotype-1 and serotype-2 (p-value = 0.02) (Table 2).

**Table 2:** Lymphocyte and platelet median counts in DENV-POS and DENV-NEG patients and according to DENV serotype among DENV-POS patients. IQ: InterQuartil interval

		Lymphocytes count (cells/mm3)		Platelets count (G/L)	
		Median (IQ)	p-value	Median (IQ)	p-value
Dengue diagnosis	DENV-NEG (n = 181)	1330 (980; 1930)	<0.001*	208.5 (163.7; 267.0)	< 0.001*
	DENV-POS (n = 94)	700 (500; 1000)		159.0 (123.0; 202.0)	
Dengue serotype	1 (n = 9)	1000 (540; 1120)	0.02^£^	168.0 (160.0; 224.0)	0.54^£^
	2 (n = 16)	820 (645; 978)		166.0 (140.5; 197.7)	
	3 (n = 27)	500 (440; 755)		155.0 (127.0; 197.0)	

*: Mann-Whitney test

£: Kruskal-Wallis test

Regarding platelet counts, it appears that despite median platelet count being significantly lower in DENV-POS patients than in DENV-NEG patients (Table 2), thrombocytopenia remained inconstant (median platelet count of 159 G/L among DENV-POS patients) and occurred mainly between the 3rd day and the 7th day post onset. Median platelet count did not vary according to DENV serotype (p = 0.54) (Table 2).

### Statistical impact of the combination of clinical picture and lymphopenia among the Dengue-suspected patients in our study

The negative predictive value (NPV) related to the combination of fever, arthralgia, lymphopenia and its specificity were analyzed. In our study, the NPV equaled 100% at Day 1 post onset of symptoms and was superior to 80% till Day 6. The specificity increased from 40% at Day 1 to 80% at Day 6 post onset of symptoms.

The NPV related to the combination of fever, arthralgia, gastro-intestinal symptoms and lymphopenia inferior to 1,000cells/mm3 from Day 1 to Day 7 post onset of symptoms was superior to 70% and its average specificity remained superior to 85%.

Moreover, among the DENV-POS cases presenting the combination of fever, arthralgia, and gastro-intestinal symptoms, 8.3% tested without lymphopenia and 91.7% with lymphopenia. In contrast, we observed the same proportion of DENV-NEG cases presenting fever, arthralgia, gastro-intestinal symptoms without and with lymphopenia (47.8% and 52.2% respectively). Among the patients presenting the combination of fever, arthralgia, and gastro-intestinal symptoms without lymphopenia, 15.3% are DENV-POS and 84.7% are DENV-NEG. Among the patients presenting the combination of fever, arthralgia, and gastro-intestinal symptoms with lymphopenia, 64.7% are DENV-POS and 35.3% are DENV-NEG.

## Discussion

Between 2011 and 2014, with 128 (36.2%) cases diagnosed among 354 suspected cases, the rate of detected Dengue cases was greater than the one observed by Houze in 2003 (12). In that retrospective study, the author described 185 cases (confirmed by serologies and/or viral cultures) between January 2000 and March 2003 found among 1,224 patients with a non-malarial fever. This increase in Dengue case declaration could be explained by the emergent epidemiological profile of Dengue, by a more exhaustive patient recruitment, but also by the improvement of biological diagnostic tests facilitating Dengue virus detection (NS1 Ag detection at the Bouffard hospital laboratory and RT-PCR assays performed by the Arboviruses NRC), resulting in an increase of case notification. However, the incidence of Dengue is definitely underestimated, due to the recruitment bias associated with the administrative accessing rules to the Bouffard hospital and to the recurrent lack of rapid diagnosis test at the laboratory (medical supply problems). Effectively, hospital consultation is mainly restricted to an affluent population, which is better protected against mosquito attacks and consults at the earliest stages of the disease. Finally, the study was restricted to symptomatic patients, with all asymptomatic patients ruled out. Dengue virus is symptomatic in only around 50% of cases [5].

With an epidemic of 12,000 cases reported by Rodier et al. [11], active surveillance of Dengue began in Djibouti in the early 1990’s. Since then, it has been circulating in an endemo-epidemic pattern. Each year between December and May, an epidemic peak is reached, due to the abundance of its vector (peak in the number of mosquitoes due to an increase in rainfall between December and May). In 2012, following the heavy spring rainfalls, an increase in the number of cases was observed until June. For the first time in Djibouti, serologic typing of dengue cases that arose during the epidemic in winter 2011–2012, confirmed DENV3 cases. An epidemic of DENV3 has been described in 2010 in Sudan [16]. Another epidemic occurred in Kenya in October 2011, with about 5,000 suspected cases, in a border district with Somalia and Ethiopia [17]. DENV1 appears to circulate in an endemic pattern. During the surveillance period of our study, no DENV4 case was diagnosed, as expected this genotype is sparsely reported in Africa [18]. However, due to the lack of a national arboviral surveillance system, Dengue circulation in the Horn of Africa is difficult to assess.

Our results highlight a higher rate of arthralgia in DENV-POS patients than in DENV-NEG patients. Despite no statistically significant association found in this study, patients presenting with gastrointestinal symptoms tended to be more likely to have Dengue. No statistical link was found between clinical signs and DENV serotypes. This could be due to a lack of sampling power. During the first week of symptoms evolution, analyzing data from the complete blood count demonstrated an early and almost constant lymphopenia in DENV-POS adult patients, which has already been described [19]. This study also demonstrated that a lymphocyte count below 1,000 cells/mm3 is associated with Dengue. It was already reported that lymphopenia is associated with severe forms of Dengue [20]. A lymphocyte count higher than 1,500 cells/mm3 was unlikely to be associated with the diagnosis of Dengue in our study. Even if platelet counts are lower in DENV-POS patients, thrombocytopenia is inconstant in DENV-POS patients on the day of diagnosis. This is due to the fact that thrombocytopenia is delayed in uncomplicated Dengue cases in adult patients [19]. Biological and clinical data collected during this study offer a direction in diagnosis for physicians working in isolated areas, suggesting that Dengue-suspected patients with arthralgia, gastrointestinal symptoms and a lymphopenia below 1,000cells/mm3 are very likely to be DENV-POS. A 10 day follow up of DENV-POS patients is recommended with paracetamol treatment until the cessation of the symptoms. Among 354 Dengue suspected cases, only 3 cases could be attributed to a known arbovirus other than Dengue leaving 63% of the non-malarial Dengue-like cases undetermined. No infection caused by Chikungunya virus was recorded during this period. However, Borgherini et al. [21] have shown that infection caused by Chikungunya virus is also associated with lymphopenia and severe thrombocytopenia. Nkoghe et al. [22] put forward that gastrointestinal symptoms are similar in Chikungunya and in Dengue infections and that in both cases lymphopenia below 1, 000 cells/mm3 can be noted. Similar symptoms may also be found in infections due to other arboviruses, such as Zika infection for example. Data of the present study may therefore help guiding the diagnosis in favor of an arbovirus, if they are not in favor of Dengue.

The 2 severe clinical cases observed during this study were associated with DENV3. There is no clear data on the link between serotypes and severity of the infection in previous studies [23,24] but severity seems to depend on a previous exposure to another serotype (Halstead's controversial theory about immunological facilitation) [25]. Moreover, retro-orbital pain and skin rash are more frequent for DENV3. Biologically, lymphopenia below 800 cells/mm3 also suggests the presence of DENV3. However, these results were not observed in previous studies [23] and should therefore be confirmed on larger numbers of patients. After sequencing these strains, we have determined that they belonged to genotype III, often associated to more severe symptomatic forms [24, 26]. Although Dengue has only recently come up in Africa [27], we expect that DENV3 will spread throughout the continent in the coming years, with more severe Dengue forms in naive countries.
